# Kikuchi–Fujimoto disease in the Eastern Mediterranean zone

**DOI:** 10.1038/s41598-022-06757-9

**Published:** 2022-02-17

**Authors:** Abdel Rahman Al Manasra, Hamzeh Al-Domaidat, Mohd Asim Aideh, Doaa Al Qaoud, Majd Al Shalakhti, Sohaib Al khatib, Jehad Fataftah, Raed Al-Taher, Mohammad Nofal

**Affiliations:** 1 Department of General Surgery and Urology, Faculty of Medicine, Jordan University of Science and Technology, P.O. Box 3030, Irbid, Jordan; 2 Department of Pathology, Faculty of Medicine, Jordan University of Science and Technology, Irbid, Jordan; 3 Department of Pediatrics, Faculty of Medicine, The Hashemite University, Zarqa, Jordan; 4 Department of Radiology, Faculty of Medicine, The Hashemite University, Zarqa, Jordan; 5 Department of Surgery, Faculty of Medicine, The University of Jordan, Amman, Jordan

**Keywords:** Diseases, Medical research, Rheumatology

## Abstract

Kikuchi–Fujimoto disease (KFD) is a rare benign and self-limiting syndrome. We aim to review cases of KFD at our institution as a rare illness in the Arab ethnic descent and to analyse reports from most countries in the East Mediterranean zone. This is a retrospective study in which the histopathology database was searched for the diagnosis of KFD. A full review of KFD patients’ medical records was done. Data regarding demographic features, clinical presentation, laboratory findings, comorbidities, and management protocols were obtained. Published KFD cases from east Mediterranean countries were discussed and compared to other parts of the world. Out of 1968 lymph node biopsies studied, 11 (0.6%) cases of KFD were identified. The mean age of patients with KFD was 32 years (4–59). 73% (8/11) were females. The disease was self-limiting in 5 patients (45%); corticosteroid therapy was needed in 4 patients (34%). One patient was treated with methotrexate and one with antibiotics. One patient died as a consequence of lymphoma. Jordanians and Mediterranean populations, especially those of Arab ethnic background, seem to have low rates of KFD. The genetic susceptibility theory may help to explain the significantly higher disease prevalence among East Asians. Early diagnosis of KFD—although challenging—is essential to reduce the morbidity related to this illness.

## Introduction

Kikuchi–Fujimoto disease (KFD), which is also known as histiocytic necrotizing lymphadenitis, is a rare cause of lymphadenopathy^1^, that was first recorded in Japan in 1972 by Kikuchi and Fujimoto^2,3^. It is an extremely rare benign, self-limited disease. The condition is also characterized by fever, night sweats, fatigue and to a lesser extent skin rash and joint pain^4^. The exact cause of KFD is still obscure. However, the acute clinical course, febrile self-limited nature, as well as the histologic changes associated with this illness, all support the theory of immune response to an infectious etiology, primarily viral agents, such as Epstein-Barr virus (EBV), human herpesviruses 6 and 8, HIV, parvovirus B19^5–7^, and Torque teno/torque teno-like minivirus (TTV/TTMV), which closely resembles the circovirus that causes necrotizing lymphadenitis in pigs. TTV/TTMV presence in patients with KFD has been confirmed with successful amplification and DNA sequencing^8,9^.

Kikuchi and Fujimoto originally identified this pathology among Japanese and other Asiatic women; the condition has since been defined worldwide in both genders and in a variety of ethnic backgrounds, with higher prevalence among females^10^. From our region (middle-east/Arabic countries), there are few scattered reports of KFD that have been published over the last 20–30 years.

In this retrospective study, we aim to review and summarize previous reports of KFD in the east Mediterranean countries and to describe the incidence as well as the characteristics of KFD among Jordanians as a Mediterranean population of Arab ethnic background. We assessed KFD in terms of clinical, laboratory and epidemiological features. Comorbid illnesses, treatment protocols and clinical outcomes were analyzed as well. To our knowledge, this is the largest descriptive analysis of KFD in Jordan.

## Results

Between 2006 and 2021, 1968 lymph node biopsies were submitted to our histopathology laboratory. Eleven cases (0.6%) had the diagnosis of KFD. Mean age of patients with KFD was 32 years (4–59). 73% (8/11) were females.

Fatigue (82%), appetite loss (81%) and fever (63%) were the most frequent presenting symptoms. On physical examination, Lymphadenopathy was the most common finding (11/11) followed by arthritis (4/11) and hepatosplenomegaly (2/11). 82% (9/11) of the patients had cervical lymphadenopathy while only 18% (2/11) had axillary lymphadenopathy. (Table 1).

**Table 1:** King Abdullah University Hospital KFD Patients’ demographic and clinical characteristics.

Variable	n	%
Age (years), mean (range)	32 (4–59)	
**Gender**		
Male	3	27
Female	8	73
**Comorbidities**		
SLE^a^	1	9
Seizures	1	9
Lymphoma	1	9
Chronic kidney disease	1	9
Cystic hygroma	1	9
**Presenting symptoms**		
Fever	7	63
Fatigue	9	81
Joint pain	3	27
Weight loss	3	27
Loss of appetite	9	81
Sweating	1	9
**Physical findings**		
Lymphadenopathy	11	100
Arthritis	4	36
Hepatosplenomegaly	2	18
Apthous ulcer	1	9
**Laboratory findings**		
Leukocytosis	7	63
Elevated ESR^c^	8	72
Anemia	3	27
Elevated liver enzymes	4	36
Positive ANA^c^	2	18
Elevated LDH^d^	6	54

^a^Systemic lupus erythromatosus.

^b^Erythrocyte sedimentation rate.

^c^Antinuclear antibodies.

^d^Lactate dehydrogenase.

37 reports of KFD in the East Mediterranean zone were retrieved, with a total of 95 patients. 15 reports (60 patients) were from Arabic countries. Lymphadenopathy (in 32/37 studies) and fever (in 20/37 studies) were the main presenting symptoms, whereas fatigue and joint pain were only occasionally reported (in 4/37 and 1/37 reports, respectively). In fact, these findings apply to both Arab and non-Arab Mediterranean countries included in Table 2.

**Table 2:** Reports of KFD^a^ from Eastern Mediterranean countries.

Country (total cases 95)	Authors	Year	Number of cases	Presenting symptoms
Arabian Gulf states (56)	**Saudi Arabia**			
	Louis et al.	2007	2	Cervical LAP^b^ and pyrexia
	Alsolami et al.	2021	1	Recurrent LAP
	Al-Maghrabi et al.	2005	15	Axillary (1) and cervical (14) LAP
	Amir et al.	2002	2	Painful cervical LAP, fever, fatigue
	**Qatar**			
	Al-Allaf et al.	2018	1	Painless right inguinal lump
	Mohamad et al.	2020	11	Painful neck swelling and fever
	Halawa et al.	2020	1	Fever, fatigue and weight loss
	Al Soub et al.	2021	1	Neck swelling, fever and nausea
	**Bahrain**			
	Al Mosawi et al.	2020	11	Prolonged fever, LAP
	**United Arab Emirates**			
	Shamsuddeen et al.	2016	1	Swelling of right side of neck
	Helal et al.	2001	10	Cervical (8) and axillary (2) LAP
Lebanon (2)	Tariq et al.	2014	1	Multiple right-sided neck swellings
	Hamdan et al.	2009	1	Cervical lymphadenopathy and neck pain
Syria (1)	Youssef et al.	2017	1	Fatigue, arthralgia, fever, night sweats, anorexia and weight loss, painful cervical lymphadenopathy
Iran (8)	Servatyari et al.	2020	1	Fever, weight loss with cervical LAP
	Baziboroun et al.	2019	1	Fever and LAP
	Taghvaei et al.	2015	1	Cervical, axillary and inguinal LAP
	Aminiafshar et al.	2013	1	Chills, fever, cervical LAP, myalgia
	Behdadnia et al.	2016	1	Persistent fever for 6 weeks
	Anikhindi et al.	2017	1	Fever of unknown origin
	Aznab et al.	2015	1	Cervical LAP and neck pain
	Jaseb et al.	2021	1	6-month history of left cervical LAP
Turkey (13)	Açoğlu et al.	2018	1	Fever, abdominal pain, and weight loss, axillary LAP
	Küçük et al.	2017	1	Weakness, joint aches, fever, loss of appetite, weight loss, night sweating symptoms
	Koybasi et al.	2003	2	Enlarged cervical lymph nodes
	Uslu et al.	2014	1	Cervical LAP, fever, maculopapular rash
	Altuntas et al.	2006	1	Prolonged fever, generalized LAP
	Yalcin et al.	2011	1	Cervical LAP, anorexia, fever
	Aydogan et al.	2006	1	Fever, fatigue and sweat, diarrhoea, cervical LAP
	Yilmaz et al.	2006	2	Cervical LAP
	Soy et al.	2007	1	N/A^c^
	Dane et al.	2009	1	Generalized LAP
	Urun et al.	2011	1	Cervical LAP
Jordan (1)	Albaramki et al.	2017	1	Prolonged fever, weight loss, hepatosplenomegaly, and generalized LAP
Greece (14)	Charalabopouls et al.	2002	4	Lymphadenitis, fever, weight loss
	Vassilakopoulos et al.	2009	9	Cervical (8) and axillary (1) LAP, fever
	Gionanlis et al.	2009	1	Fever, cervical LAP

^a^Kikuchi–Fujimoto disease.

^b^Lymphadenopathy.

^c^Data not available.

When compared to other ethnic groups, tender lymphadenitis (50%) and fever (43%) were, similarly; the most frequent clinical findings in studies from East Asia (Taiwan) and China^4,11,12^, while Joint pain and hepatosplenomegaly were much less encountered in the Asian ethnicity. Reports from western countries, which may still include up to 20% of patients from Asian backgrounds^13^, also showed that lymphadenopathy and fever represent the classic hallmarks of the disease^3^.

Some of our patients from Jordan had other comorbidities including systemic lupus erythematosus (SLE) and chronic kidney disease (1/11), B cell non-Hodgkin’s lymphoma (1/11), epilepsy (1/11), migraine headache (1/11) and cystic hygroma (1/11). (Table 1).

In Laboratory work up, elevated erythrocyte sedimentation (ESR) rate (72%) (Range of abnormal findings: 27-more than 100, normal reference Values: 0–20), leukocytosis (63%) (Range of abnormal readings: 11.5 × 10^3^–17 × 10^3^, normal reference values: 4–11 × 10^3^) and elevated lactate dehydrogenase (LDH) (54%) (Range of abnormal readings: 282–1789, normal reference values: 240–480) were the most common findings. (Table 1).

Most of our patients had self-regression of the disease without any targeted treatment (5/11) (45%). Treatment with oral corticosteroids was attempted in 4 (34%) patients (prednisolone dosage 0.5 mg/kg per day, duration range: 2 weeks–9 months). All corticosteroids’ therapy group recovered completely without relapse. Oral antibiotics (Amoxicillin and clavulanate potassium; Beta-lactamase inhibitor) were prescribed in two cases, as well.

## Discussion

In this study, we investigated the prevalence rate of KFD among Jordanian patients who underwent lymph node biopsy at our center. Our results showed that out of each thousand lymph node biopsies, 6 were diagnosed with KFD (0.6%), which is a comparable rate to others reported in non-Asian communities.

Mediterranean populations of Arab ethnic background seem to have similar rates. Reported numbers from the gulf region might be slightly affected by non-Arab ethnicities included in the studies. In Kuwait^14^, between 2005 and 2009, a study reviewed 2,369 fine needle aspirations (FNA) of patients with lymphadenopathy. Of these, 76 (3.2%) patients were diagnosed with KFD or were suggestive of KFD, 51 of the 76 (67%) KFD patients were non-Kuwaiti, primarily from the Indian Subcontinent and South-East Asia. In Saudi Arabia, a study included 2500 lymph node biopsies, revealed KFD in only 15 (0.6%) patients^15^. Another report from Saudi Arabia by Kutty MK et al. recognized only 5 cases of KFD in 920 lymph node biopsies (0.5%)^16^.

A-14-patient case series from Qatar by Al Soub et al.^17^, represented a KFD incidence rate of approximately 1.6% from 900 lymph node biopsies; Almost half (43%) of KFD cases in this series were of non-Qatari nationalities.

Similarly, large data from other Mediterranean ethnicities such as Turki^4^, Iranian^18^, and Greek^19^, are still lacking. The published small-volume reports may indicate low incidence rate. In addition, review of these reports revealed clinical characteristics that correspond with data from other Mediterranean populations. Table 2 summarizes the main reports of KFD from Eastern Mediterranean countries.

Studies from Asia disclosed 10–40 times higher incidence rates of KFD, especially among female patients with cervical lymphadenopathy, which seemed to be the main presenting sign. In a retrospective study of 147 Korean patients who had enlarged (≥ 1 cm in diameter) cervical lymph nodes for a duration > 1 week, 51 (34.7%) had KFD. All patients in this study had core needle biopsies obtained under ultrasound guidance^11^. In their analysis of published literature, Kucukardali and colleagues showed that nearly 50% (166/330) of reported KFD cases between 1991 and 2005 originated in East Asia and the Far-East, with about 36% being from Taiwan, which had the majority of reported cases^4^.

There is no clear explanation for the lower incidence of KFD among Arab and other non-Asian ethnicities. However, some studies raised the theory of genetic susceptibility that makes east-Asians at higher risk to become affected. In one study by Tanaka and colleagues^20^, they performed DNA typing of human leukocyte antigens (HLA) class II genes (HLA-DR, -DQ, and -DP), and found that frequencies of DPA1*01 and DPB1*0202 allele in HLA class II genes were significantly higher among KFD patients; this allele is relatively frequent in Asians (e.g., Korean 9.9%, Japanese 4.5%) when compared to Caucasians (e.g., French 0.4%, Italian 0.8%), and Arabs (Lebanese 2.97%^21^)^22,23^. It is also unclear if environmental triggers play a role in the pathogenesis of this illness.

Although KFD is considered a benign condition, the significant morbidity with frequent hospitalization may be attributed to mistaken diagnosis with other lymphoproliferative disorders, connective tissue diseases or infectious etiologies. Multiple other factors may also contribute to the increased morbidity of KFD; these include either disease-linked complications or concurrent other systemic illnesses. Complications that have been described varied from potentially fatal manifestations, such as disseminated intravascular coagulopathy (DIC)^24,25^, abrupt heart failure, KFD-triggered hemaphagocytic syndrome, and polymyositis with respiratory failure^26–28^, to less extensive and regional implications similar to lymphedema and cutaneous eruption^29,30^. Moreover, some reports described the occurrence of other conditions simultaneously with KFD, such as Hashimoto thyroiditis^31,32^, aseptic meningitis (only 4 cases reported)^33,34^ and Still’s disease^35^; these may contribute to the increased morbidity of the disease.

The diagnosis of KFD is challenging. It relies on a combination of clinical and histopathologic features. Histologically, the disease is evaluating in different stages represented by three subtypes; the early proliferative type, the necrotizing type and the xanthomatous type. The necrotizing type is the most common and characterized by the presence of non-neutrophilic necrotizing foci surrounded by mononuclear cells and show abundant karyorrhectic debris. The early type is characterized by the presence of predominant immunoblastic paracortical infiltrate admixed with histiocytes. The healing phase of KFD is mostly represented by the presence of foamy histiocytes and referred to as xanthomatous type^36,37^.

Cases with abundant immunoblastic proliferation may be mistaken for high grade lymphoma. However, the presence of abundant histiocytic infiltrate, lack of complete lymph node effacement by B-lymphocytes Immunohistochemical markers and loss of clonality proof by B-surface immunoglobulin arrangement and T-cell receptor gene rearrangement, both favour the diagnosis of KFD^36^.

The mechanism by which KFD is linked to other autoimmune diseases remains unknown. Some researchers hypothesized that KFD was a T-cell mediated response to antigen stimuli in genetically susceptible individuals based on immunostains and histological findings^3^.

Affected patients with KFD may need to be followed for years due to the possibility of association with systemic lupus erythematosus. In addition, recurrences of Kikuchi disease can occasionally continue for many years^38^. Early detection and treatment of KFD to control active inflammation is crucial, owing to the presumptive reduction in the risk of consequent limb swelling and permanent lymphedema.

KFD is nominated as a self-limiting disease in the majority of cases. However, supportive treatment, with non-steroidal anti-inflammatory drugs (sDIASN) is used for many patients to alleviate associated symptoms, such as fever, lymphadenitis-induced pain or arthralgia. Since etiology of this disease is still uncertain, definitive treatment remains challenging. In multiple studies corticosteroids were used for severe forms of disease (not responding to NSAIDS, prolonged course), relapsing lymphadenitis, or in patients with KFD manifestations (as listed above)^39^. Other reports described successful use of hydroxychloroquine and intravenous immunoglobulin (IVIG) for intractable disease^38,40^.

Our study has some limitations. First, it is a retrospective study. Second, the sample size of patients with KFD was relatively small to evaluate risk factors with statistical significance. In spite of these limitations, findings in this study should expand awareness of KFD, contribute to better understanding of clinical behaviour of this illness and highlight the importance of formulating a solid consensus regarding diagnosis and treatment protocols.

In conclusion, KFD may still be under-diagnosed in our part of the world, likely due to the non-specific mixed symptoms at presentation as well as to the lack of awareness among general practitioners and health care providers. Early diagnosis with tissue biopsy and treatment is essential to reduce associated morbidity.

## Materials and methods

### Study design

This is a retrospective study in which we identified patients diagnosed with KFD between January 2002 and January 2021. All patients included were diagnosed at King Abdullah University Hospital (KAUH). Jordan. The histopathology department’s database was searched for KFD during this period. Biopsies were collected locally at KAUH and at affiliated hospitals, and then submitted to KAUH. Full review of patients’ medical records was done. Patients were also contacted for follow up and verification whenever necessary. Data regarding demographic features, clinical presentation, laboratory findings, comorbidities and management protocols were obtained.

Work up for lymphadenopathy was driven by clinical evaluation. The following investigations were obtained as needed: complete blood count (CBC) and peripheral blood smear, Lactate dehydrogenase (LDH), Kidney function test, Liver function test and urine analysis, ESR and CRP. Screening for TB (sputum for Acid-Fast Bacillus (AFB), PPD testing), HIV (human immunodeficiency virus), and specific titers for Epstein-Barr virus, cytomegalovirus (CMV) and *Toxoplasma* species were rarely considered because patients presented almost always with regional lymph node enlargement.

Imaging studies included chest radiography, computerized tomographic (CT) scan for chest and/or abdomen, abdominal and cervical ultrasonography.

To retrieve reports of KFD in the Eastern Mediterranean region, the keywords (KFD, necrotizing lymphadenitis, histiocytic, Mediterranean, Middle East, Arab, and the individual name of each of the listed states in Table 1, were searched through PubMed or Medline, Embase Scopus, and Ovid databases. Full texts of the studies were obtained and year of publication, number of cases, main presenting symptoms were recorded.

### Statistical analysis

IBM SPSS v.20.0 (Chicago, IL, USA) software was used for statistical analysis.

### Inclusion criteria

All adult or pediatric Jordanian patients with biopsy proven KFD disease, who were diagnosed at King Abdullah university hospital, and affiliated centers, were included.

### Ethics and confidentiality

The study was assessed and approved by the Institutional Review Board at King Abdullah University Hospital and by the Committee of Research on Human Subjects at the Jordan University of Science and Technology. (Non-funded Research Grant No: 20210141). We protected Patients’ confidentiality in accordance with declaration of Helsinki provisions. Informed consent was waived by the ethics Institutional Review Board at King Abdullah University Hospital and by the Committee of Research on Human Subjects at the Jordan University of Science and Technology that approved our study Procedures.

For tissue diagnosis, most patients (10/11) in this series had open lymph node biopsy. Only one patient underwent ultrasound guided Tru-Cut® axillary lymph node biopsy.

### Histopathology

**Figure 1:**
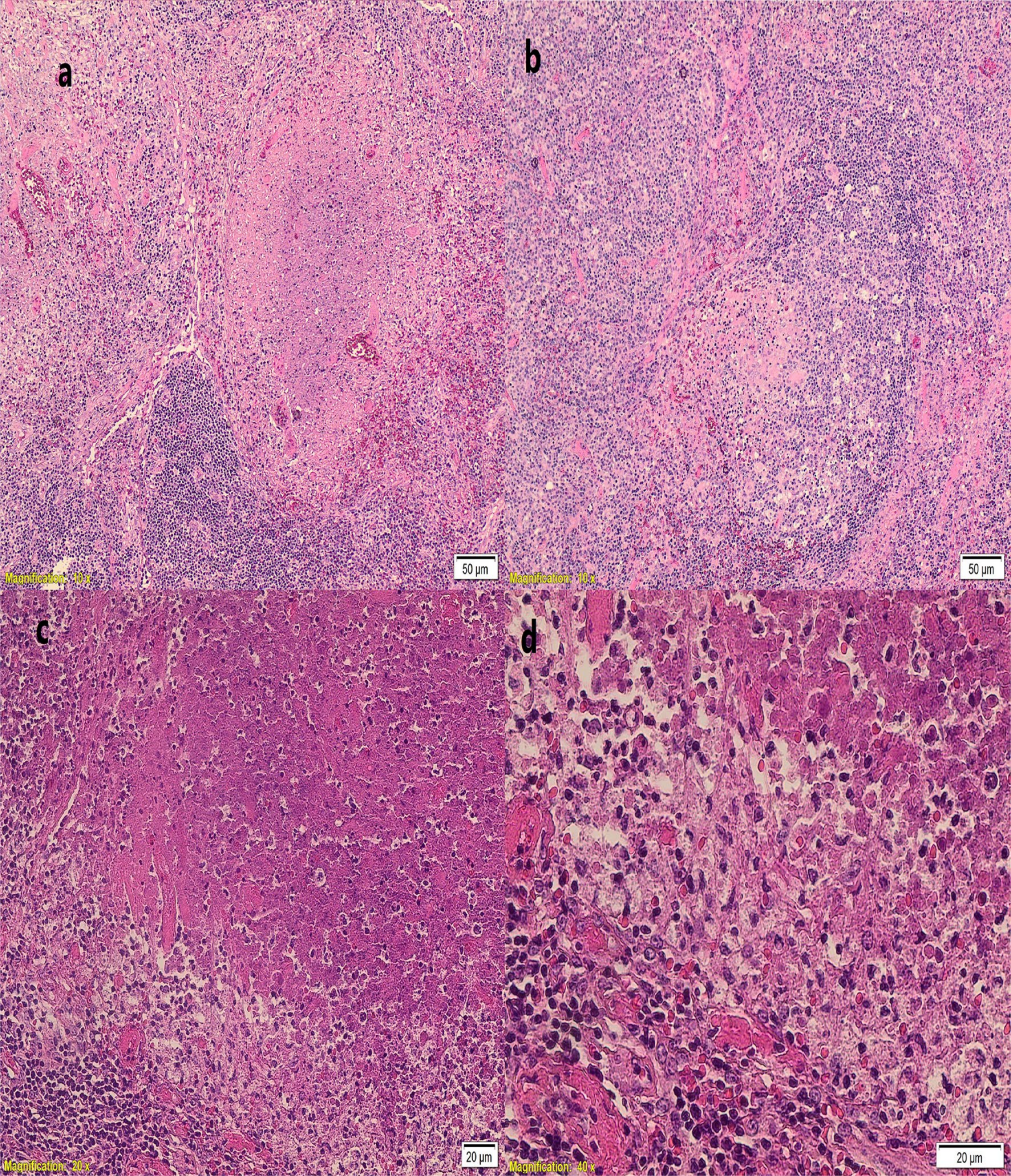
Lymph node from a patient with KFD. (**a**,**b**) The lymph node architecture is effaced by areas of confluent necrosis. (**c**,**d**) Higher magnification show karyorrhectic debris within necrotic foci which are surrounded by mononuclear cells including histiocytes and immunoblasts.

Lymph node biopsies were analyzed and reported by hematopatholohist. The histologic diagnosis of KFD was defined based on the presence of patchy areas of paracortical non-suppurative necrosis with the presence of abundant karyorrhectic debris. The necrotic areas are surrounded by mononuclear cells predominantly histiocytes including crescentic histiocytes. (Fig. 1).

Immunohistochemical studies including CD20, CD3, Cd4, CD8, and CD68 were performed on selected cases whenever indicated.

## Data Availability

The datasets generated during and/or analysed during the current study are available from the corresponding author on reasonable request.
