# Author Correction: Kikuchi–Fujimoto disease in the Eastern Mediterranean zone

**DOI:** 10.1038/s41598-022-11109-8

**Published:** 2022-04-28

**Authors:** Abdel Rahman Al Manasra, Hamzeh Al-Domaidat, Mohd Asim Aideh, Doaa Al Qaoud, Majd Al Shalakhti, Sohaib Al khatib, Jehad Fataftah, Raed Al-Taher, Mohammad Nofal

**Affiliations:** 1 Department of General Surgery and Urology, Faculty of Medicine, Jordan University of Science and Technology, P.O. Box 3030, Irbid, Jordan; 2 Department of Pathology, Faculty of Medicine, Jordan University of Science and Technology, Irbid, Jordan; 3 Department of Pediatrics, Faculty of Medicine, The Hashemite University, Zarqa, Jordan; 4 Department of Radiology, Faculty of Medicine, The Hashemite University, Zarqa, Jordan; 5 Department of Surgery, Faculty of Medicine, The University of Jordan, Amman, Jordan

Correction to: *Scientific Reports* 10.1038/s41598-022-06757-9, published online 17 February 2022

The original version of this Article contained an error in Affiliations 3 and 4, which were incorrectly given as ‘Department of Radiology, Faculty of Medicine, Hashemite University, Zarqa, Jordan’ and ‘Department of Pediatrics, Faculty of Medicine, Hashemite University, Zarqa, Jordan’. The correct affiliations are listed below:

Department of Pediatrics, Faculty of Medicine, The Hashemite University, Zarqa, Jordan.

Department of Radiology, Faculty of Medicine, The Hashemite University, Zarqa, Jordan.

The original Article has been corrected.

